# Saikosaponin‐d impedes hippocampal neurogenesis and causes cognitive deficits by inhibiting the survival of neural stem/progenitor cells via neurotrophin receptor signaling in mice

**DOI:** 10.1002/ctm2.243

**Published:** 2020-12-21

**Authors:** Tingting Qin, Ziqiao Yuan, Jiayu Yu, Xinxin Fu, Xueyang Deng, Qiang Fu, Zhanqiang Ma, Shiping Ma

**Affiliations:** ^1^ Department of Pharmacology of Chinese Materia Medica China Pharmaceutical University Nanjing China; ^2^ Jiangsu Key Laboratory of Drug Screening China Pharmaceutical University Nanjing China; ^3^ Qinba Traditional Chinese Medicine Resources Research and Development Center AnKang University Ankang China

**Keywords:** neurogenesis, neurotoxicity, p75^NTR^, saikosaponin‐d, TrkB

## Abstract

Neural stem/progenitor cells (NPCs) are multipotent stem cells in the central nervous system. Damage to NPCs has been demonstrated to cause adverse effects on neurogenesis and to contribute to neurological diseases. Our previous research suggested that saikosaponin‐d (SSd), a cytostatic drug belonging to the bioactive triterpenoid saponins, exhibited neurotoxicity by inhibiting hippocampal neurogenesis, but the underlying mechanism remained elusive. This study was performed to clarify the role of SSd in cognitive function and the mechanism by which SSd induced damage to hippocampal neurogenesis and NPCs. Our results indicated that SSd caused hippocampus‐dependent cognitive deficits and inhibited hippocampal neurogenesis by reducing the numbers of newborn neurons in mice. RNA sequencing analysis revealed that SSd‐induced neurotoxicity in the hippocampus involved neurotrophin receptor‐interacting MAGE (NRAGE)/neurotrophin receptor interacting factor (NRIF)/p75^NTR^‐associated cell death executor (NADE) cell signaling activated by the p75 neurotrophin receptor (p75^NTR^). Mechanistic studies showed that a short hairpin RNA targeting p75^NTR^ intracellular domain reversed SSd‐increased NRAGE/NRIF/NADE signaling and the c‐Jun N‐terminal kinase/caspase apoptotic pathway, subsequently contributing to the survival of NPCs, as well as cell proliferation and differentiation. The addition of recombinant brain‐derived neurotrophic factor (BDNF) ameliorated the SSd‐induced inhibition of BDNF/Tyrosine kinase receptor B (TrkB) neurotrophic signaling, but did not affect SSd‐activated pro‐BDNF/p75^NTR^ signaling. Moreover, the SSd‐induced elevation of cytosolic Ca^2+^ concentration was responsible for damage to NPCs. The extracellular Ca^2+^ chelator ethylene glycol‐bis(2‐aminoethylether)‐N,N,N',N'‐tetraacetic acid (EGTA), rather than the intracellular Ca^2+^ chelator 1,2‐bis(2‐aminophenoxy)ethane‐N,N,N',N'‐tetraacetic acid tetrakis(acetoxymethyl ester) (BAPTA/AM), attenuated SSd‐induced cytosolic Ca^2+^ dysfunction and SSd‐disordered TrkB/p75^NTR^ signaling. Overall, this study demonstrated a new mechanism for the neurotoxic effect of SSd, which has emerging implications for pharmacological research of SSd and provides a better understanding of neurotoxicity induced by cytostatic drugs.

## Graphical abstract

Saikosaponin‐d induced cognitive dysfunction by inhibiting the survival of hippocampal neural stem/progenitor cells. This neural damage is through the activation of p75^NTR^ cell death signaling and inhibition of TrkB signaling, engaged by disordered BDNF pathway dependent on the dysregulation of cellular calcium homeostasis. These results present a neurological base for saikosaponin‐d‐induced neurotoxicity and provide a better understanding of neurotoxic effects induced by the cytostatic drug.

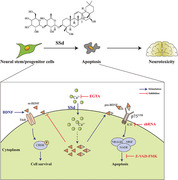

## 1 INTRODUCTION

Neural stem/progenitor cells (NPCs) are multipotent stem cells in the central nervous system (CNS) with self‐renewal ability and are sufficient to provide a large population of brain cells through differentiation into neurons or glia. Neurogenesis involves complex processes in which NPCs undergo proliferation, differentiation into neurons, migration, and integration into functional neural networks in the brain.^1^ As it said, there are two neurogenic regions occurring neurogenesis in the adult mammalian brain, one of which is the dentate gyrus (DG) in the hippocampus.^2^ Adult hippocampal neurogenesis has attracted a great deal of interest due to its unique relation to numerous CNS disorders. The effects of pathological stimuli in the hippocampus have been suggested to influence the process of neurogenesis and lead to neurological disorders. Although there is still controversy regarding the extent and relevance of adult neurogenesis in humans, there is accumulating evidence that adult neurogenesis persists in rodents and is closely related to cognitive function.^3^, ^4^

The neurotrophins and their receptors, which are abundantly expressed in the hippocampus, are crucial for maintaining synaptic plasticity and promoting the survival and differentiation of the nervous system.^5^ Several neurotrophins have been shown to determine adult neurogenesis of NPCs, among which brain‐derived neurotrophic factor (BDNF) was suggested to play a major role in the DG of the hippocampus.^6^ This interest was further supported by previous studies indicating that BDNF signaling increased the proliferation of NPCs, the survival of newborn neurons, and promoted hippocampal neurogenesis.^7^, ^8^, ^9^ Like many biological polypeptides, BDNF is initially synthesized as a pro‐peptide, pro‐neurotrophin (pro‐BDNF), which is then cleaved to release the mature protein neurotrophin (m‐BDNF).^10^ It has been demonstrated that m‐BDNF promotes neuronal survival and differentiation through initiation of neurotrophic signaling by binding to tyrosine receptor kinase B (TrkB).^11^ In contrast, pro‐BDNF has been suggested to induce apoptosis by interacting with the p75 neurotrophin receptor (p75^NTR^), which is a receptor for the neurotrophins belonging to the tumor necrosis factor receptor superfamily.^12^, ^13^ Upon activated by pro‐neurotrophins, p75^NTR^ induces apoptosis through c‐Jun N‐terminal kinase (JNK) induction and caspase activation.^14^ The intracellular death domain of p75^NTR^, which undergoes γ‐secretase‐mediated intramembrane cleavage leading to the release of a 20 kDa intracellular domain (ICD), has been suggested to be the structural determinant involved in the regulation of different signaling.^15^, ^16^ In studies examining the mechanism underlying the receptor signal transduction, numerous proteins have been shown to bind to ICD, several of which, including neurotrophin receptor interacting factor (NRIF), p75^NTR^‐associated cell death executor (NADE), and neurotrophin receptor‐interacting MAGE (NRAGE), were identified to be associated with apoptosis signaling.^17^, ^18^, ^19^ Pharmacological experiments have established that p75^NTR^ can regulate different biological activities, such as neuronal death and/or survival, axonal growth, and synaptic plasticity.^20^, ^21^ Indeed, p75^NTR^ plays an important role in integrating the pro‐apoptotic function of pro‐neurotrophins and the neurotrophic effects of mature neurotrophins. Accordingly, pro‐BDNF neurotoxic signaling related to p75^NTR^ has been reported to be correlated with neuropathological processes by contributing to hippocampal neurons apoptosis.^13^ Emerging evidence reveals that NPCs express both TrkB and p75^NTR^, and there is a constraint relationship between p75^NTR^ signaling and TrkB signaling in regulating hippocampal neurogenesis,^22^ however, few studies explore the involving mechanisms.

Saikosaponin‐d (SSd) is a bioactive triterpenoid saponin extracted from the traditional Chinese medicine Radix Bupleuri, a herbal drug that is commonly used to treat chronic hepatic inflammation and viral hepatitis in China and other Asian countries.^23^ SSd is also a typical toxic saikosaponin known as a cytostatic drug for the potent cytotoxicity toward various types of cancer cells and primary cells. The increase in cell membrane permeability through nonselective calcium (Ca^2+^) ionophores has been suggested to account for the cytotoxicity of SSd.^24^ Data from toxicology studies have indicated the potential risk of SSd‐induced hepatotoxicity and nephrotoxicity.^25^ Furthermore, recent research demonstrated that SSd exhibited neurotoxic effects by inhibiting adult hippocampal neurogenesis in mice,^26^ although the underlying mechanism remained unclear. The present study was performed to determine the effects of SSd on cognitive function and adult hippocampal neurogenesis in mice, as well as the effects of SSd on primary hippocampal NPCs. The underlying biological signaling was evaluated by RNA sequencing (RNA‐Seq) analysis, and the mechanism related to neurotrophin receptor signaling was examined both in vivo and vitro. Moreover, as the xenobiotic‐induced disruption of intracellular Ca^2+^ homeostasis is capable of inducing pathological processes and triggering neurotoxicity, we further examined whether Ca^2+^ homeostasis contributed to the cytotoxicity of SSd on NPCs and determined how neurotrophin receptor signaling affected hippocampal neurogenesis to establish clinical prevention strategies.

## 2 MATERIALS AND METHODS

### 2.1 Drugs and reagents

SSd (CAS number: 20874‐52‐6, ≥ 98%) was purchased from Nanjing Zelang Medical Technology Co., Ltd. (Nanjing, China). Accutase solution and B‐27 Supplement were obtained from Sigma‐Aldrich Co., Ltd. (St. Louis, MO) and Thermo Fisher Scientific Inc., (Waltham, MA) respectively. EGF, bFGF, and mouse recombinant BDNF were purchased from Peprotech Inc. (Cranbury, NJ). EGTA (ethylene glycol‐bis(2‐aminoethylether)‐N,N,N',N'‐tetraacetic acid), 1,2‐bis(2‐aminophenoxy)ethane‐N,N,N',N'‐tetraacetic acid tetrakis(acetoxymethyl ester) (BAPTA/AM), and Z‐VAD‐FMK were obtained from Apexbio Technology LLC (Houston, TX). Trizol reagent and Reverse transcription kit used for qPCR were supplied by Vazyme Biological Technology Co., Ltd. (Nanjing, China).

**Highlights**

1. Saikosaponin‐d impeded hippocampal neurogenesis and caused cognitive deficits in mice.
2. Saikosaponin‐d inhibited the survival of hippocampal neural stem/progenitor cells through disrupted pro‐BDNF/p75NTR and BDNF/TrkB signaling.
3. Intracellular Ca2+ dysregulation was associated with SSd‐induced neurotoxic effects.

Primary antibodies against BrdU (#ab8152), SOX2 (#ab79351), Doublecortin (DCX, #ab18723), NeuN (#ab177487), BDNF (#ab108319), MAP2 (#ab11267), glial fibrillary acidic protein (GFAP) (#ab7260), and p‐CREB (Ser133) (#ab32096) were obtained from Abcam plc (Cambridge, MA). Antibodies against p‐JNK (Tyr183/185) (#9255), JNK (#9258), BAX (#2772), BCL2 (#3498), cleaved caspase‐3 (#9661), p‐TrkB (Tyr706/707) (#4621), and TrkB (#4603) were supplied by Cell Signaling Technology Co., Ltd. (Danvers, MA). Antibody against p75^NTR^ (#G3231) was purchased from Promega Biotech Co., Ltd. (Madison, WI). Antibodies against NADE (#PA5‐20076), Nestin (#MA1‐110), and NRIF (#OSZ00002G) were purchased from Thermo Fisher Scientific Inc. Antibody against NRAGE (#22053‐1‐AP), CREB (#12208‐1‐AP), and β‐actin (#20536‐1‐AP) were obtained from Proteintech Group Inc. (Chicago, IL).

### 2.2 Animals

C57BL/6J mice (male, 7‐8 weeks) were supplied by Vital River Laboratory (Beijing, China). All the animals were kept in cages under standardized environmental conditions at 25 ± 2°C temperature with photoperiod (12 hours of light followed by 12 hours of the dark cycle). All food and water were free to access. Animal experiments involved were approved by the Ethics Committee of China Pharmaceutical University and the Laboratory Animal Management Committee of Jiangsu Province.

### 2.3 Drug treatment

**FIGURE 1:**
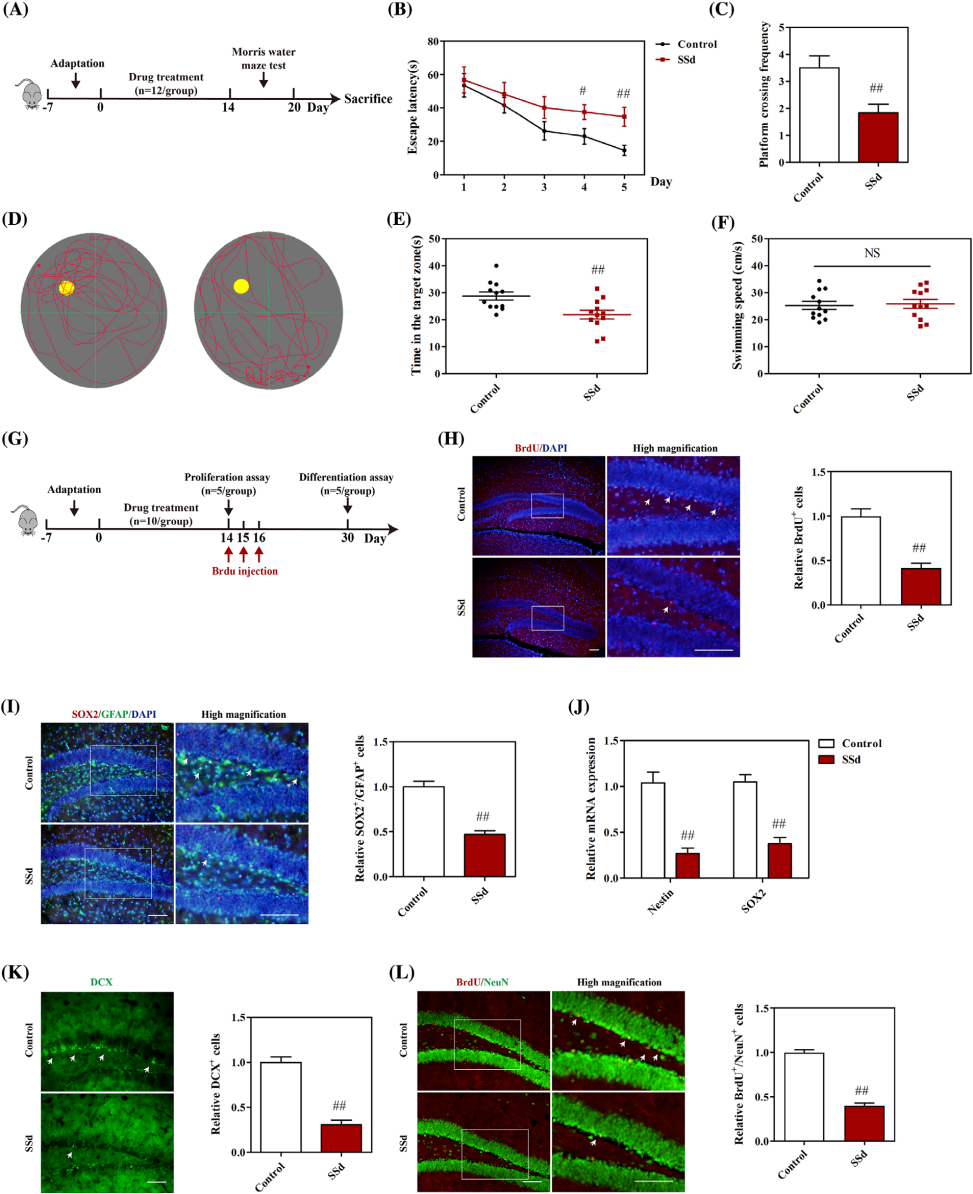
SSd caused cognitive dysfunction and impaired hippocampal neurogenesis in mice. A, The timeline of the experimental procedure. B, The escape latency of mice during the training and test days (n = 12). C, Quantitative analysis of the frequency of mice crossing the platform on the test day (n = 12). D, The swimming tracks of mice on the test day. E, Quantitative analysis of time in the target zone of mice on the test day (n = 12). F, The swimming speed of mice during the test (n = 12). G, The timeline of the experimental procedure. H, Immunofluorescence analysis of BrdU (red) and quantitative analysis of BrdU^+^ cells in the DG of the hippocampus (n = 4). Scale bar 100 μm. I, Immunofluorescence analysis of SOX2 (red) and GFAP (green) and quantitative analysis of SOX2^+^/GFAP^+^ cells in the DG of the hippocampus (n = 4). Scale bar 50 μm. J, The mRNA expressions of Nestin and SOX2 were detected by qPCR in the hippocampus (n = 6). K, Immunofluorescence analysis of DCX (green) and quantitative analysis of DCX^+^ cells in the hippocampus (n = 4). Scale bar 50 μm. L, Immunofluorescence analysis of BrdU (red) and NeuN (green) and quantitative analysis of BrdU^+^/NeuN^+^ cells in the DG of the hippocampus (n = 4). Scale bar 50 μm. Data are expressed as mean ± SEM. Abbreviation: NS, not significant versus the control. ^#^ *P *< .05 versus the control. ^##^ *P *< .01 versus the control

Animals were treated with SSd as described previously.^27^ To determine the effects of SSd on cognitive function, mice were randomly divided into two groups (n = 12/group) and received drug treatment for 14 consecutive days: control group (saline, i.g.) or SSd group (16 mg/kg, i.g.). After treatment, mice were subjected to the Morris water maze test and then sacrificed for further analysis. To explore the effects of SSd on adult hippocampal neurogenesis, mice were randomly divided into two groups (n = 10/group) and treated as described above. BrdU (Sigma, St. Louis, MO) was prepared by being dissolved in normal saline for neuronal labeling. The dose of BrdU used was demonstrated previously to have no toxic effects on the animals.^28^ On day 14 of treatment, five mice in each group were injected with BrdU (100 mg/kg, i.p.), and the tissues were harvested 2 hours later for analysis of cell proliferation. The remaining five mice in each group received BrdU (100 mg/kg, i.p.) six times over 3 days (twice daily) and were sacrificed 14 days later for cell survival analysis. The experimental procedure is shown schematically in Figures 1A and 1G .

### 2.4 Morris water maze test

The Morris water maze test was performed to assess hippocampus‐dependent learning and memory function.^29^ Briefly, a circular tank was divided into four parts with a platform submerged in the water in the center of one of the quadrants. Animals were randomly placed into the maze from four points during the 4 training days. Animals were guided to and allowed to remain on the platform for 15 seconds if they failed to find the platform within 90 seconds. Twenty‐four hours after the last training session, the platform was removed, and the animals were allowed to swim for 90 seconds. A video tracking program was used to record the swimming behaviors of mice for further analysis.

### 2.5 Immunohistochemistry

Immunohistochemistry and cell quantification were carried out as described previously.^30^ Generally, mice were anesthetized with sodium pentobarbital. After perfusion of phosphate buffered saline (PBS) and paraformaldehyde (PFA), the brains were post‐fixed in PFA and dehydrated in 10%, 20%, and 30% sucrose solution before cutting into sections 40 μm thick. The tissue sections were washed with PBS and 0.2% Triton X‐100 in PBS (PBST), blocked with 5% BSA, followed by sequential incubation with the primary and second antibodies. For BrdU staining, DNA was denatured with 1 N HCl and 2 N HCl, and borate buffer was used for neutralization. Tissue sections were then washed with PBS, blocked with 5% BSA, and incubated with the primary and second antibodies as described above. To analyze the long‐term survival of NPCs, immunostaining was performed for SOX2/GFAP, DCX, and BrdU/NeuN. Fluorescence images were obtained using an inverted microscope (IX53; Olympus, Tokyo, Japan). The cells were quantified as previously described.^30^, ^31^ Briefly, the positive cells were determined in the subgranule zone and granule cell layer of the hippocampus. All sections were encoded and examined by observers blinded to the group allocation. One of every six sections was counted. ImageJ software (NIH, Bethesda, MD) was used to measure the surface of sections. The stereological cells were calculated by the volume of the dentate granule cell layer (the sum of traced areas multiplied by the thickness of slices). The number of positive cells in the SSd group was then normalized relative to the control.

### 2.6 qPCR analysis and RNA‐Seq

Total RNA was isolated from the hippocampus with Trizol reagent according to the manufacturer's instructions. The RNA was then quantified to the same concentration and converted to cDNA using Transcription Kit. Subsequently, cDNA samples were performed on Stepone Plus using the Power SYBR Green Master Mix with GAPDH as the internal control. The sequences of the forward and reverse primers are listed in Table 1.

**TABLE 1:** The primer sequences used for real‐time quantitative PCR assay in mice

Gene	Forward primer	Reverse primer
GAPDH	CTTTGGCATTGTGGAAGGGCTC	GCAGGGATGATGTTCTGGGCAG
Nestin	AGGAGAAGCAGGGTCTACAGAG	AGTTCTCAGCCTCCAGCAGAGT
SOX2	AACGGCAGCTACAGCATGATGC	CGAGCTGGTCATGGAGTTGTAC

RNA‐Seq was performed after the quantification and quality assurance of RNA samples. The libraries were sequenced on the Illumina HiSeq 4000 platform (Novogene Technology, Beijing, China). FastQC was used to assess the quality of reads and filters. Gene set enrichment analysis (GSEA) was conducted to identify the related biological function enrichment. Reactome gene set database was used for the gene set collection, and the downloaded dataset was imported using the GSEA software 1000 times for each analysis was repeated in the process according to the default weighted enrichment statistical method. Gene sets were identified as significantly enriched when the false discovery rate‐adjusted *P*‐value < .05.

### 2.7 TUNEL assay

Coronal brain sections were prepared as the immunohistochemistry staining described. TUNEL staining was performed according to the manufacturer protocol. Cells with TUNEL‐positive and 4',6‐diamidino‐2‐phenylindole dihydrochloride (DAPI) staining were identified as apoptotic cells. Fluorescence images were obtained with the IX53.

### 2.8 Primary NPCs isolation and culture

Primary NPCs were isolated from the brains of embryos at embryonic day (E) 14.5 as described previously.^32^, ^33^ Briefly, embryonic brains were extracted from mice and placed in D‐Hank's balanced salt solution (Gibco, Grand Island, NY). The hippocampus was dissected by gentle pipetting followed by seeding into DMEM/F‐12 media containing B‐27 Supplement (2%), bFGF (20 ng/mL), and EGF (20 ng/mL) in 100‐mm cell culture dishes (NEST Biotechnology, Wuxi, China). The cells were then cultured in a humidified 37°C, 5% CO_2_ incubator to form neurospheres, and half of the medium was replaced with fresh medium after 3 days. The neurospheres were passaged every 6 days with a final density of 5 × 10^5^/mL. Further analyses were performed using the cells at passage three.

### 2.9 Cell viability assay

The effects of SSd on cell viability were determined by the 3‐(4, 5‐dimethylthiazol‐2‐yl)‐2, 5‐diphenyl tetrazolium bromide (MTT) method. The dissociated NPCs were seeded into the 96‐well plate pre‐coated with poly‐L‐lysine (PLL, Sigma). Cells were then treated with different concentrations of SSd for 24 hours. After that, cells were incubated with 20 μL of MTT (0.5 mg/mL) at 37°C for 4 hours. To explore the effects of apoptosis on SSd‐altered cell viability, cells were pre‐treated with Z‐VAD‐FMK (50 μm) 30 minutes before SSd incubation. The precipitate was dissolved in 200 μL dimethylsulfoxide and detected with the microplate reader at 570 nm.

### 2.10 Neurosphere assay

Neurosphere cell proliferation was detected using well growth neurosphere on the third‐generation. Neurospheres were cultured in the 6‐well plate and incubated with SSd for 24 hours. The effects of BDNF on neurosphere survival were conducted using the incubation of mouse recombinant BDNF in the absence or presence of SSd. The concentration of BDNF was 40 ng/mL as the previous study reported.^34^ Images were obtained from a microscope.

### 2.11 EdU assay

NPCs were seeded into the 48‐well plate pre‐coated with PLL. After treatment with SSd for 12 hours, 5‐ethynyl‐2′‐deoxyuridine (EdU, 10 μm) was added to the cells and incubated for a further 12 hours. Subsequently, cells were fixed and permeabilized by 4% PFA and PBST, respectively. A Cell‐light TM EdU kit (Beyotime, Shanghai, China) was used for visualization of EdU, and cell nuclei were stained with DAPI. Fluorescence images were obtained with the IX53.

### 2.12 Differentiation assay

NPCs were seeded into the 24‐well plate pre‐coated with PLL. The differentiation of NPCs was performed using a differentiation medium consisting of DMEM/F‐12 supplemented with 1% FBS. NPCs were cultured in differentiation medium with or without SSd for 7 days followed by fixation with 4% PFA and washing with PBST. After blocking with 5% BSA for 2 hours, cells were incubated with primary antibodies to microtubule‐associated protein 2 (MAP2) and GFAP overnight at 4°C. The cells were then incubated with Alex Fluor‐488/594‐conjugated secondary antibodies at 37°C for 1 hour. DAPI was used to counterstain the cell nuclei. Fluorescence images were captured using the IX53.

### 2.13 Hoechst 33342 assay

Hoechst labeling was used to detect apoptotic nuclei as previous research.^35^ In brief, NPCs were seeded into the 48‐well plate pre‐coated with PLL. Then NPCs were treated with SSd for 24 hours. Z‐VAD‐FMK was pretreated 30 minutes before SSd incubation as above described. After that, cells were gently washed with PBS followed by incubating with Hoechst 33342 (Sigma). Images were obtained using the Olympus IX53 fluorescent microscope.

### 2.14 shRNA transfection

To silence p75^NTR^ ICD expression, a lentiviral short hairpin RNA (shRNA) vector targeting the mouse p75^NTR^ ICD (NM_033217.3) was created by GenePharma Co., Ltd. (Shanghai, China). The RNA targeting sequences of p75^NTR^ ICD were designed according to the previous research.^36^, ^37^ The knockdown of p75^NTR^ ICD was confirmed at the mRNA level by qPCR. Neurospheres were digested and transfected with a control shRNA (negative control, sh‐NC) or the targeting shRNA at a multiplicity of infection (MOI) of 10 for 12 hours. Then cells were cultured with the normal NPCs medium for 72 hours. After further treatment, cells were subjected to Western blot, EdU, and differentiation assays as above described.

### 2.15 Measurement of Ca^2+^ concentration

The Ca^2+^ assay kit (Abcam) was used to measure the hippocampal Ca^2+^ concentration of mice according to the manufacturer's instructions. Generally, hippocampus tissues were homogenized with a sonicator using calcium assay buffer. After centrifuged, supernatant was collected and incubated with chromogenic reagent for 10 minutes at room temperature. Then measure output on a microplate reader at 575 nm. The concentration of Ca^2+^ was calculated and normalized by the standard wells.

The Ca^2+^ concentration of NPCs was visualized by Fluo‐4 AM staining as a fluorescent Ca^2+^ probe. Neurospheres were exposed to SSd incubation with or without EGTA (extracellular Ca^2+^ chelator, 1 mM) and BAPTA/AM (intracellular Ca^2+^ chelator, 2 μm) for 24 hours, respectively. Then cells were digested and washed with Hank's balanced salt solution. After incubating with a working solution containing 0.02% Pluronic F‐127 (Thermo Fisher) and Fluo‐4 AM (2 μm) at 37°C for 45 minutes, cells were centrifugated. PBS was used to wash cells, and the nucleus was stained with DAPI. Fluorescence images were captured using the IX53.

### 2.16 Western blot analysis

Hippocampus tissues and primary NPCs were prepared with RIPA lysis buffer. Protein concentrations were measured using a BCA protein assay kit (Beyotime). A total of 30 μg protein was separated by 12% ‐ 15% sodium dodecylsulphate (SDS)‐PAGE (polyacrylamide gel electrophoresis) and transferred onto polyvinylidene difluoride (PVDF) membranes followed by incubation in blocking buffer for 2 hours. The membranes were then incubated with primary antibodies overnight at 4°C and with appropriate secondary antibodies for 2 hours at room temperature. Signals were detected by chemiluminescence and quantified using an imaging system (Bio‐Rad, Hercules, CA).

### 2.17 Statistical analysis

Statistical analyses were conducted using GraphPad Prism 6 (GraphPad Software, San Diego, CA). Data are presented as the mean ± SEM. The statistical significance of differences was examined by two‐tailed Student's *t*‐test or one‐way analysis of variance followed by Tukey's multiple comparisons. In all analyses, *P* < .05 was taken to indicate statistical significance.

## 3 RESULTS

### 3.1 SSd caused cognitive dysfunction and impaired hippocampal neurogenesis in mice

The effects of SSd on cognitive function in mice were evaluated using the Morris water maze test. The detailed experimental procedure is shown in Figure 1A. The time taken to find the platform was increased for SSd‐treated mice in comparison with controls on both the training and probe days (Figure 1B). Moreover, the mice treated with SSd showed poorer performance compared to the controls with regard to the residence time in the target zone (Figures 1D and 1E). The platform crossing frequency of SSd‐treated mice was also lower than that of the controls (Figure 1C). Interestingly, there was no significant difference in swimming speed between the SSd‐treated and control mice (Figure 1F), indicating that SSd caused cognitive dysfunction without affecting physical performance.

The levels of neurogenesis in the hippocampus have been suggested to be associated with hippocampus‐dependent learning and memory.^38^ Therefore, we examined the effects of SSd on hippocampal neurogenesis by evaluating cell proliferation and the long‐term survival of neuroblasts in the DG of mice. The results of BrdU immunostaining showed that there were fewer BrdU^+^ cells in the DG of SSd‐treated mice compared with the controls (Figure 1H), indicating that SSd inhibited cell proliferation in the hippocampus. As the radial glia‐like cells (RGLs) represent the NPC population with the ability to give rise to proliferating progenitor cells and generate neuroblasts in neural niches,^39^ we further assessed the numbers of GFAP^+^/SOX2^+^ cells. Mice in the SSd treatment group showed significantly fewer GFAP^+^/SOX2^+^ co‐labeled cells in the subgranular zone compared to the controls (Figure 1I). Moreover, qPCR analysis indicated that the levels of SOX2 and Nestin mRNAs were markedly decreased in the SSd group in comparison with the controls (Figure 1J), suggesting that SSd inhibited the self‐renewal capacity of NPCs in the hippocampus of mice. The long‐term survival of neuroblasts was examined by immunostaining for neuronal markers. As shown in Figure 1K, the numbers of DCX^+^ cells were markedly reduced in SSd‐treated mice compared with the controls. Meanwhile, mice treated with SSd showed significantly reduced numbers of BrdU^+^/NeuN^+^ co‐labeled cells than the controls (Figure 1L). Taken together, these observations indicated that SSd impaired hippocampal neurogenesis and suppressed the survival of newborn neurons in the hippocampal DG.

### 3.2 SSd activated NRAGE/NADE/NRIF cell apoptotic signaling in the hippocampus

**FIGURE 2:**
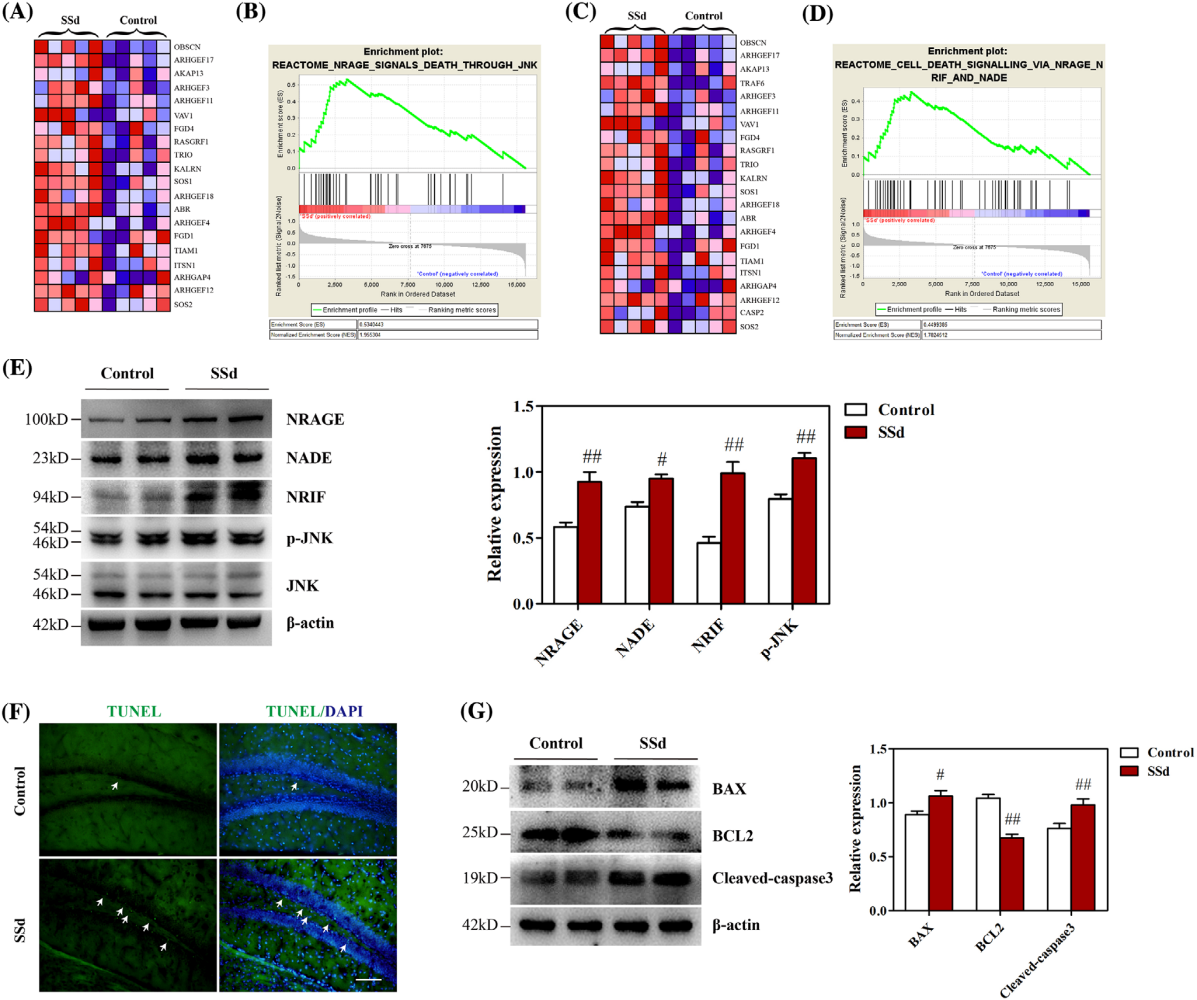
SSd activated NRAGE/NADE/NRIF cell apoptotic signaling in the hippocampus. A, A heat map of the NRAGE pathway‐related genes shows a strong correlation in SSd‐treated mice (n = 5). B, GSEA profiles of the correlation between SSd‐treated mice and NRAGE signaling. C, A heat map of the NRAGE/NRIF/NADE pathway‐related genes shows a strong correlation in SSd‐treated mice (n = 5). D, GSEA profiles of the correlation between SSd‐treated mice and NRAGE/NRIF/NADE signaling. The upper part plots the enrichment scores for each gene, and the lower part of the plot shows the value of the ranking metric moving down the list of ranked genes. E, Western blot analysis of NRAGE/NRIF/NADE signaling in the hippocampus and quantitative analysis of the levels of NRAGE, NRIF, NADE, and p‐JNK (n = 4). F, Hippocampal cell apoptosis was evaluated by TUNEL staining in the DG (n = 4). Scale bar 50 μm. G, Western blot analysis of the apoptotic proteins in the hippocampus and quantitative analysis of the levels of BAX, BCL2, cleaved caspase‐3 (n = 4). Data are expressed as mean ± SEM. ^#^ *P *< .05 versus the control. ^##^ *P *< .01 versus the control

To determine the mechanism by which SSd impaired hippocampal neurogenesis, RNA‐Seq analysis was performed in the hippocampus of mice. GSEA was performed to explore the functional relationships among the gene products. Gene sets corresponding to the regulation of cell apoptosis, including NRAGE/JNK and NRAGE/NRIF/NADE signaling, were significantly altered in SSd‐treated mice compared with the controls, and this was suggested to be a functional association (Figures 2A‐2D). NRAGE/NRIF/NADE signaling plays a crucial role in the survival of various cells in the nervous system.^17^, ^18^, ^19^ It has been suggested that these three proteins act as bifunctional switches for cell survival or apoptosis. Previously, NRAGE, NRIF, and NADE were shown to have diverse signaling potential throughout murine embryogenesis.^40^, ^41^ To confirm whether this signaling involved SSd‐mediated neurotic effects, the expression levels of related proteins were examined. As shown in Figure 2E, western blotting analysis indicated that the levels of NRAGE, NADE, and NRIF, as well as phosphorylated JNK, were significantly higher in the SSd‐treated mice than in the controls, which was consistent with the results of gene analysis. Furthermore, the levels of apoptosis in the DG of the hippocampus were evaluated. TUNEL staining indicated elevated numbers of apoptotic cells in the DG of SSd‐treated mice compared with the controls (Figure 2F). Notably, the administration of SSd also altered the levels of apoptosis‐related proteins, including BAX, BCL2, and cleaved caspase‐3 (Figure 2G). These results suggested that SSd activated NRAGE/NADE/NRIF signaling and induced apoptosis in the hippocampus.

### 3.3 SSd exhibited cytotoxicity in primary hippocampal NPCs through NRAGE/NADE/NRIF apoptotic signaling

**FIGURE 3:**
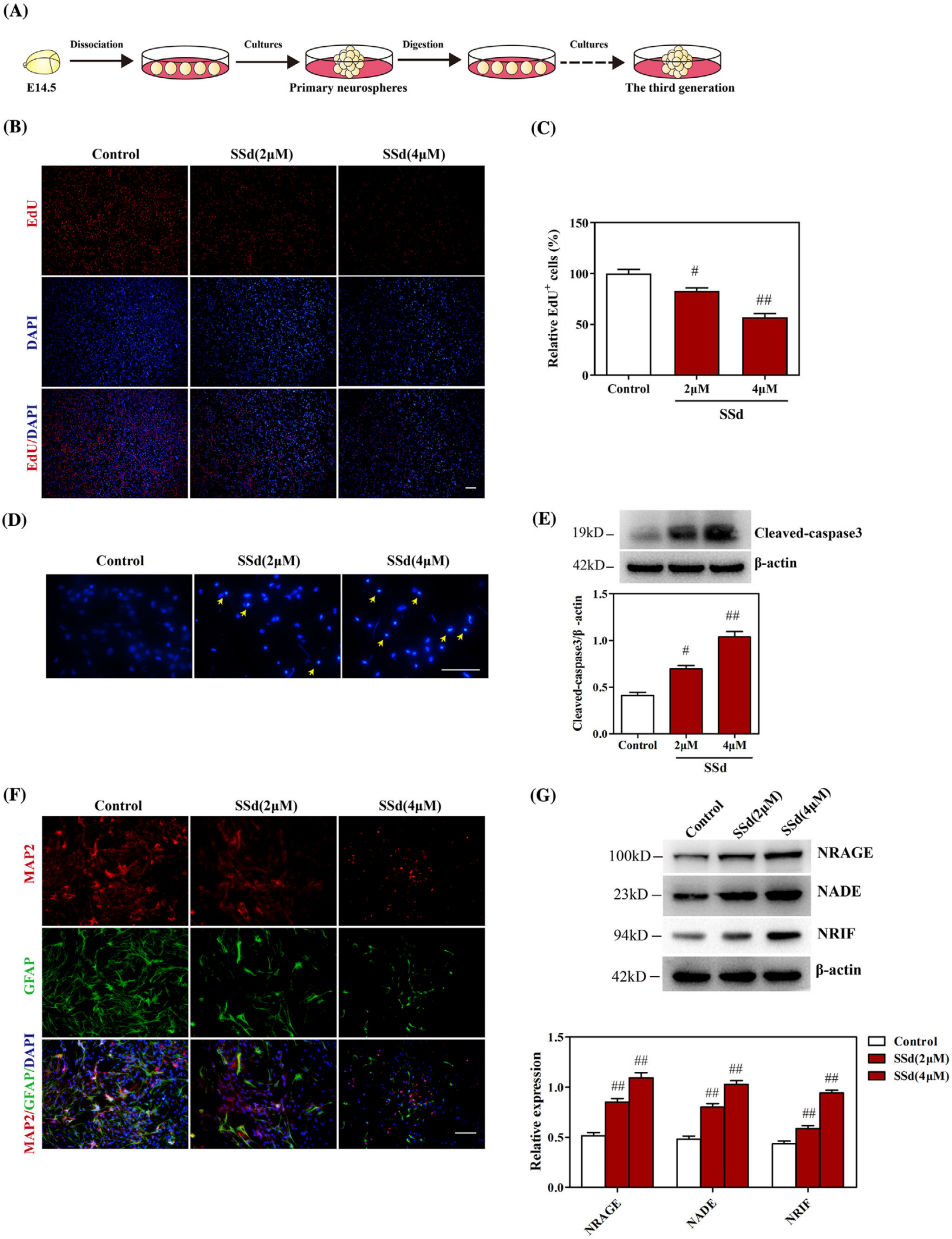
SSd exhibited cytotoxicity in primary hippocampal NPCs through NRAGE/NADE/NRIF apoptotic signaling. A, The procedure of the cultured primary hippocampal NPCs. B and C, Effect of SSd on cell proliferation and quantitative analysis of the levels of EdU^+^ cells. The NPCs were treated with SSd for 24 hours, and cell proliferation was measured using the EdU kit. Scale bar 100 μm. D, Effect of SSd on apoptosis of NPCs. The NPCs were treated with SSd for 24 hours, and cell apoptosis was detected using Hoechst 33342 staining. Scale bar 25 μm. E, Western blot analysis of cleaved caspase‐3 and quantitative analysis of the levels of cleaved caspase‐3. The neurospheres were treated with SSd for 24 hours and harvested for western blot analysis. F, Effect of SSd on cell differentiation of NPCs. The NPCs were incubated with the medium for differentiation and treated with SSd for 7 days followed by immunofluorescence analysis for MAP2 (red) and GFAP (green). Scale bar 50 μm. G, Effect of SSd on NRAGE/NRIF/NADE signaling in NPCs and quantitative analysis of the levels of NRAGE, NRIF, and NADE. Data are expressed as mean ± SEM. The results are representative of three independent experiments. ^#^ *P *< .05 versus the control. ^##^ *P *< .01 versus the control. Abbreviation: NS, not significant versus the control

The effects of SSd on primary hippocampal NPCs were investigated to explore further how it affected hippocampal neurogenesis. MTT assay showed that SSd inhibited the viability of NPCs in a dose‐dependent manner (Figure S1A), with an EC50 of ∼5.13 μm (4.78 ‐ 5.52 μm, 95% confidence interval). Similarly, SSd was shown to suppress the growth of neurospheres (Figure S1B). The results of EdU analysis showed that the numbers of EdU‐positive cells were reduced after exposure to SSd, indicating that SSd inhibited the proliferation of NPCs (Figures 3B and 3C). Hoechst 33342 staining indicated that exposure to SSd led to apoptosis of NPCs (Figure 3D), which was further confirmed by western blotting analysis indicating that SSd markedly increased the expression cleaved caspase‐3 (Figure 3E). Meanwhile, the caspase inhibitor, Z‐VAD‐FMK, was shown to attenuate SSd‐induced apoptosis of NPCs, reverse SSd‐induced increased cleaved caspase‐3 level, and improve the viability of NPCs (Figures S1C–S1E), indicating that SSd reduced the survival of NPCs through the apoptotic pathway. Differentiation analysis indicated that SSd further inhibited the cell differentiation morphology of NPCs (Figure 3F). Moreover, as shown in Figure 3G, SSd significantly increased the levels of NRAGE, NRIF, and NADE in NPCs, consistent with the results in mice. Taken together, these results indicated that SSd exerted cytotoxic effects on NPCs by suppressing their survival and inducing apoptosis through NRAGE/NADE/NRIF signaling.

### 3.4 SSd regulated NRAGE/NRIF/NADE signaling in NPCs by inducing activation of p75^NTR^ ICD

**FIGURE 4:**
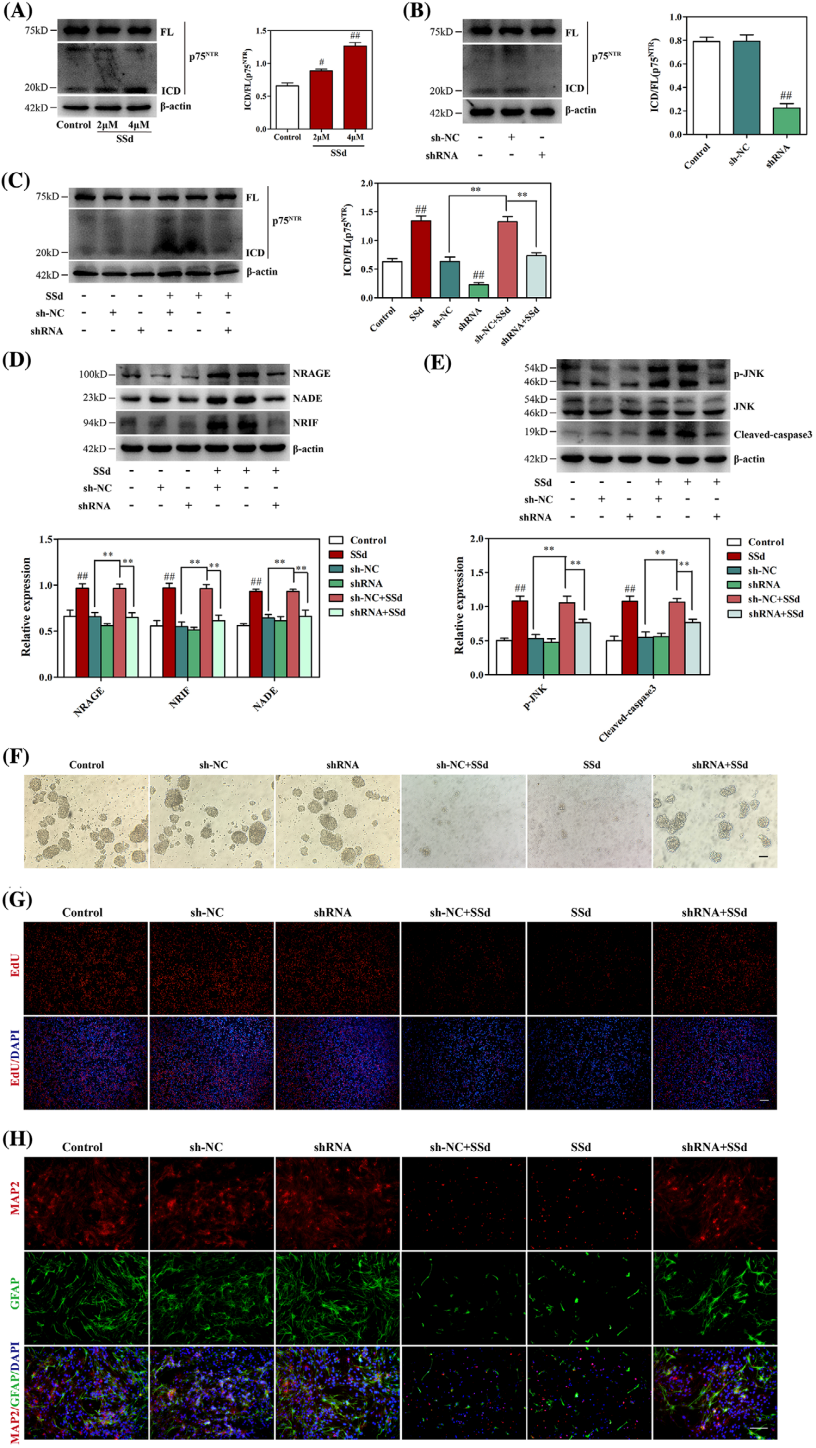
SSd regulated NRAGE/NRIF/NADE signaling in NPCs by inducing activation of p75^NTR^ ICD. A, Western blot analysis of the effect of SSd on p75^NTR^ activation in NPCs and quantitative analysis of the levels of p75^NTR^ ICD. B, Effect of p75^NTR^ ICD shRNA on the inhibition of p75^NTR^ ICD expression and quantitative analysis of the levels of p75^NTR^ ICD. The neurospheres were incubated with p75^NTR^ ICD shRNA or sh‐NC for 12 hours, then cultured with the normal medium for 72 hours, followed by western blot analysis. C, Effect of p75^NTR^ ICD shRNA on SSd‐activated p75NTR and quantitative analysis of the levels of p75^NTR^ ICD. D, Effect of p75^NTR^ ICD shRNA on SSd‐induced NRAGE/NADE/NRIF signaling and quantitative analysis of the levels of NRAGE, NADE, and NRIF. E, Effect of p75^NTR^ ICD shRNA on SSd‐induced apoptotic signaling and quantitative analysis of the levels of p‐JNK and cleaved caspase‐3. F, Effect of p75^NTR^ ICD shRNA on SSd‐induced neurospheres growth. Scale bar 100 μm. The neurospheres were exposed to SSd for 24 hours after the p75^NTR^ ICD shRNA or sh‐NC transfection, followed by western blot analysis and bright‐field analysis. G, Effect of p75^NTR^ ICD shRNA on SSd‐inhibited cell proliferation in NPCs. The NPCs were exposed to SSd for 24 hours after the p75^NTR^ ICD shRNA or sh‐NC transfection, and cell proliferation was detected by the EdU kit. Scale bar 50 μm. H, Effect of p75^NTR^ ICD shRNA on SSd‐induced cell differentiation of NPCs. After transfected with p75^NTR^ ICD shRNA or sh‐NC, the NPCs were incubated with differentiation medium for 7 days in the absence or presence of SSd followed by immunofluorescence analysis for MAP2 (red) and GFAP (green). Scale bar 50 μm. Data are expressed as mean ± SEM. The results are representative of three independent experiments. ^#^ *P *< .05 versus the control. ^##^ *P *< .01 versus the control. ^**^ *P *< .01 versus the indicated group

Previous studies have shown that NRAGE, NADE, and NRIF are crucial downstream modulators of p75^NTR^, which could trigger the p75^NTR^‐dependent apoptotic pathway.^42^ The findings outlined above indicated that SSd activated NRAGE/NRIF/NADE apoptotic signaling, which prompted us to examine further whether p75^NTR^ signaling was involved in the SSd‐induced cytotoxicity against NPCs. Western blotting analysis showed no significant changes in full‐length (FL) p75^NTR^ expression, whereas the expression of p75^NTR^ ICD was markedly increased by SSd treatment in a dose‐dependent manner leading to an increase in the ICD/FL ratio in NPCs (Figure 4A). To examine whether SSd inhibited the survival of NPCs via activation of p75^NTR^‐dependent apoptotic signaling, a shRNA targeting p75^NTR^ ICD was transfected into NPCs to knock down the expression of p75^NTR^ ICD. As shown in Figure 4B, the p75^NTR^ ICD shRNA with MOI of 10 exhibited the highest transfection efficiency against the expression of p75^NTR^ ICD in NPCs compared with the controls, which was used in the subsequent experiments. SSd promoted the expression of p75^NTR^ ICD and increased the levels of cell death signaling proteins, including NRAGE, NADE, and NRIF, while these effects were largely reversed by transfection with p75^NTR^ ICD shRNA (Figures 4C and 4D). Moreover, the effects of SSd on the levels of p‐JNK and cleaved caspase‐3 were significantly diminished in p75^NTR^ ICD shRNA‐transfected NPCs (Figure 4E), suggesting that prevention of p75^NTR^ activation could block NRAGE/NRIF/NADE apoptotic signaling, thus subsequently relieving SSd‐induced cell apoptosis in NPCs. In neurosphere assay, SSd inhibited the growth of neurospheres, and this effect was markedly ameliorated by transfection with p75^NTR^ ICD shRNA (Figure 4F). In addition, EdU analysis indicated that p75^NTR^ ICD shRNA reversed the SSd‐inhibited proliferation of NPCs (Figure 4G) and further attenuated SSd‐suppressed cell differentiation morphology (Figure 4H). Taken together, these observations indicated that the prevention of p75^NTR^ signaling facilitated the survival of NPCs in the presence of SSd and further contributed to the proliferation and differentiation of NPCs.

### 3.5 SSd prevented TrkB receptor signaling in NPCs by disrupting the expression of pro‐BDNF and m‐BDNF

**FIGURE 5:**
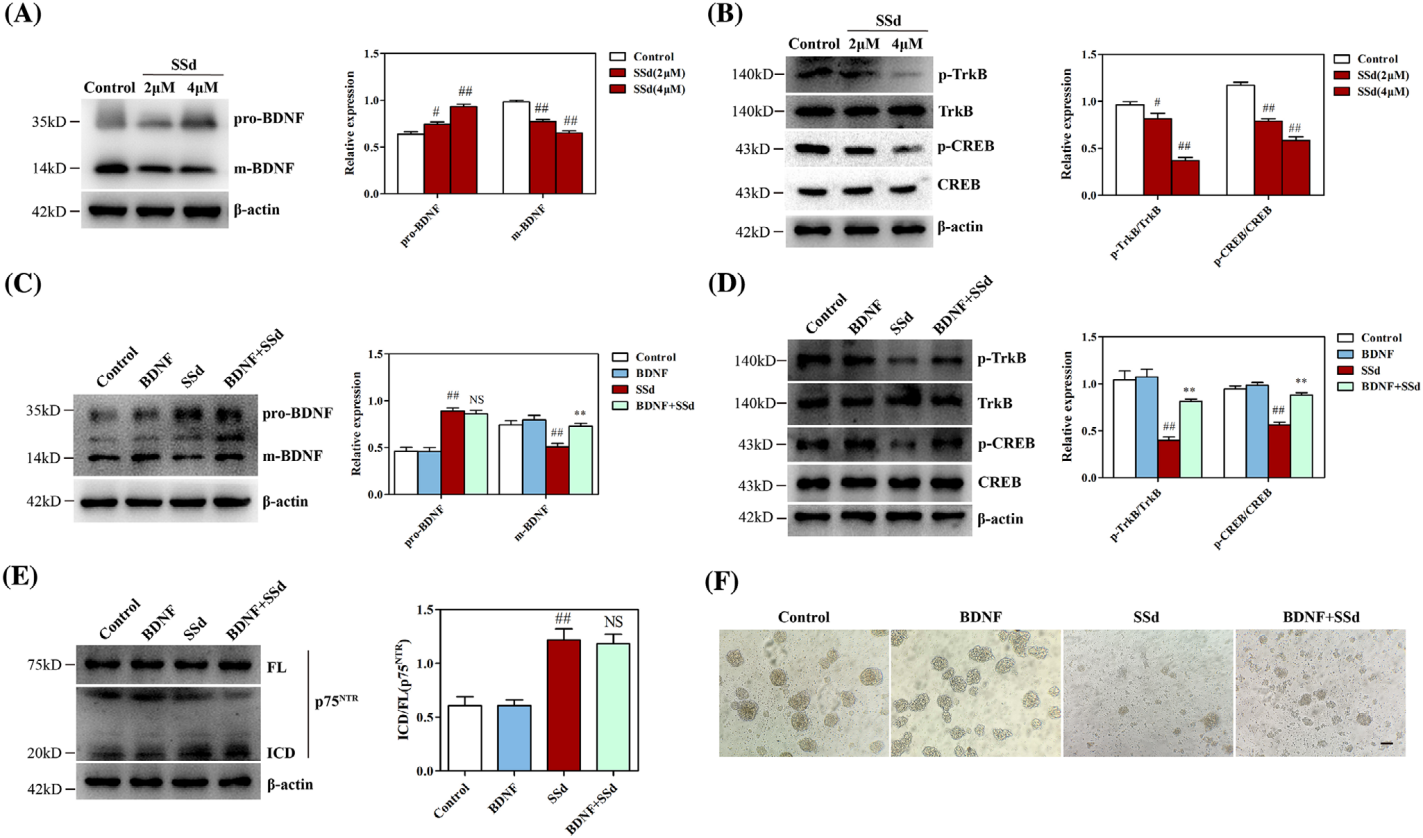
SSd prevented TrkB receptor signaling in NPCs by disrupting the expression of pro‐BDNF and m‐BDNF. A, Western blot analysis for the effect of SSd on pro‐BDNF/m‐BDNF in NPCs and quantitative analysis of the levels of pro‐BDNF and m‐BDNF. B, Western blot analysis for the effect of SSd on TrkB signaling in NPCs and quantitative analysis of the levels of p‐TrkB and p‐CREB. C, Western blot analysis of the effect of BDNF on SSd‐disordered BDNF signaling in NPCs and quantitative analysis of the levels of m‐BDNF and pro‐BDNF. D, Western blot analysis of the effect of BDNF on SSd‐inhibited TrkB/CREB signaling in NPCs and quantitative analysis of the levels of p‐TrkB and p‐CREB. E, Western blot analysis of the effect of BDNF on SSd‐activated p75^NTR^ signaling and quantitative analysis of the levels of p75^NTR^ ICD. F, Effect of BDNF on SSd‐inhibited neurospheres growth. Scale bar 100 μm. The neurospheres were exposed to SSd for 24 hours in the absence or presence of BDNF, followed by western blot and bright‐field analysis. Data are expressed as mean ± SEM. The results are representative of three independent experiments. ^#^ *P *< .05 versus the control. ^##^ *P *< .01 versus the control. ^**^ *P *< .01 versus SSd. Abbreviations: NS, not significant versus SSd

p75^NTR^ was suggested to be effectively activated by pro‐neurotrophins, while only mature neurotrophins activate TrkB. These two neurotrophin receptors are abundantly expressed in the neurogenic niches of the brain and are involved in regulating neurogenesis via BDNF signaling.^43^ In the present study, the administration of SSd markedly increased the level of pro‐BDNF protein expression and reduced the level of m‐BDNF protein expression in NPCs (Figure 5A), suggesting that the pathological status of BDNF signaling may be associated with SSd‐induced activation of p75^NTR^ signaling. It has been reported that p75^NTR^ and Trk receptors show a reciprocal relationship due to their opposing functions.^44^ As the expression of m‐BDNF was diminished by SSd, we further explored TrkB neurotrophic signaling. The results showed that exposure to SSd resulted in reduced levels of p‐TrkB and p‐CREB in NPCs compared to controls (Figure 5B), indicating that SSd disrupted the expression of pro‐BDNF and m‐BDNF, which may result in further disruption of p75^NTR^ signaling and TrkB signaling. As an important neurotrophic factor, BDNF contributes to cell survival during hippocampal neurogenesis.^9^ BDNF was reported to increase the growth of neurospheres formed by NPCs and the survival of NPCs,^34^ and the above findings suggest that SSd may have affected p75^NTR^ signaling and TrkB signaling by disordered BDNF signaling. To explore this issue, NPCs were treated with recombinant BDNF in the presence or absence of SSd. As shown in Figure 5C, the addition of BDNF reversed the SSd‐induced reduction of m‐BDNF level, but had no effect on the level of pro‐BDNF. Moreover, recombinant BDNF reversed the SSd‐induced decreases in expression of p‐TrkB and p‐CREB in NPCs (Figure 5D). Nevertheless, BDNF treatment did not affect the levels of p75^NTR^ ICD in NPCs treated with SSd (Figure 5E), indicating that p75^NTR^ signaling was also activated after the addition of recombinant BDNF in the presence of SSd. Remarkably, neurosphere assays revealed that recombinant BDNF only partially ameliorated the inhibition of neurosphere growth in the presence of SSd (Figure 5F). These observations indicated that the cytotoxicity of SSd against NPCs was mediated both through BDNF/TrkB signaling and pro‐BDNF/p75^NTR^ signaling.

### 3.6 Dysregulation of Ca^2+^ homeostasis was involved in SSd‐mediated p75^NTR^ and TrkB receptor signaling in NPCs

**FIGURE 6:**
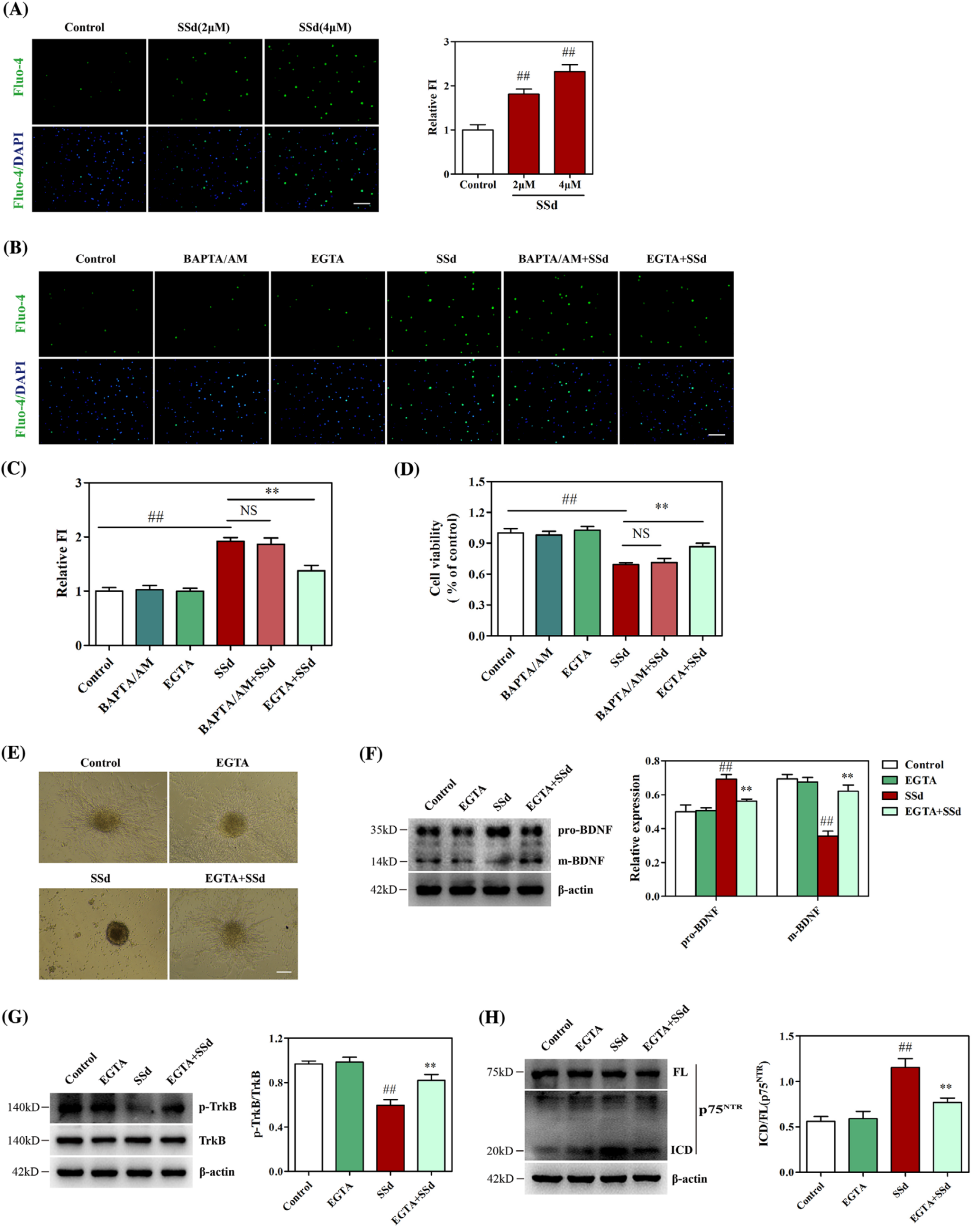
Dysregulation of Ca^2+^ homeostasis was involved in SSd‐mediated p75^NTR^ and TrkB receptor signaling. A, Effect of SSd on [Ca^2+^]_i_ of NPCs and quantitative analysis of the levels of FI. The neurospheres were digested after treated with SSd for 24 hours, and the [Ca^2+^]_i_ was detected by Fluo‐4 AM staining. B and C, Effect of EGTA and BAPTA/AM on SSd‐elevated [Ca^2+^]_i_ and quantitative analysis of the levels of FI. The neurospheres were exposed to SSd in the absence or presence of EGTA and BAPTA/AM for 24 hours, and the [Ca^2+^]_i_ was detected by Fluo‐4 AM staining. D, Effect of EGTA and BAPTA/AM on SSdinduced cell viability in NPCs. The NPCs were exposed to SSd in the absence or presence of EGTA and BAPTA/AM for 24 hours, and cell viability was measured by MTT assay. E, Effect of EGTA on SSd‐induced neurosphere growth. The neurospheres were seeded into the 6‐well plate pre‐coated with PLL and treated with EGTA in the absence or presence of SSd for 24 hours. Images were obtained from the microscope. F, Effect of EGTA on SSd‐induced BDNF signaling in NPCs and quantitative analysis of the levels of pro‐BDNF and m‐BDNF. G, Effect of EGTA on SSd‐induced TrkB signaling in NPCs and quantitative analysis of the levels of p‐TrkB. H, Effect of EGTA on SSd‐induced activation of p75^NTR^ ICD in NPCs and quantitative analysis of the levels of p75^NTR^ ICD. The neurospheres were exposed to SSd for 24 hours in the absence or presence of EGTA followed by western blot analysis. Data are expressed as mean ± SEM. Scale bar 50μm. The results are representative of three independent experiments. ^##^ *P *< .01 versus the control. ^**^ *P *< .01 versus SSd. Abbreviation: NS, not significant versus SSd

Ca^2+^ dynamics are known to influence various aspects of neurogenesis during cell proliferation, migration, differentiation, survival, and apoptosis.^45^ As SSd has been reported to be a potent Ca^2+^ ionophore, we examined whether dysregulation of Ca^2+^ homeostasis was involved in the neurotoxic effects of SSd on NPCs. Fluo‐4 AM is a fluorescent Ca^2+^ probe, the fluorescence intensity (FI) of which was used to represent the cellular concentration of Ca^2+^ ([Ca^2+^]_i_) in NPCs. As shown in Figure 6A, SSd significantly increased the FI in NPCs after 24‐hour incubation, indicating that SSd induced [Ca^2+^]_i_ overload in NPCs. To determine whether SSd‐induced cellular Ca^2+^ overload was dependent on extracellular Ca^2+^ influx or intracellular Ca^2+^ release, NPCs were incubated with the chelators EGTA and BAPTA/AM, respectively, in the absence or presence of SSd. EGTA markedly abolished [Ca^2+^]_i_ increase, whereas BAPTA/AM had no significant effect on SSd‐induced [Ca^2+^]_i_ elevation (Figures 6B and 6C), indicating that the SSd‐induced elevation of [Ca^2+^]_i_ in NPCs was from the extracellular environment. Moreover, as the [Ca^2+^]_i_ overload is suggested to be a characteristic event involved in cell apoptosis, the effects of EGTA and BAPTA/AM on cell viability were further examined. EGTA reversed SSd‐induced inhibition of cell viability, while BAPTA/AM did not abolish the decrease in cell viability due to SSd treatment (Figure 6D). Neurosphere assay showed that SSd induced the apoptosis of NPCs and inhibited the growth of neurospheres, which were largely reversed by EGTA (Figure 6E), indicating that the elevated cytoplasmic Ca^2+^ level was related to the SSd‐induced decrease of NPC survival. It has been established that cytosolic Ca^2+^ levels can regulate BDNF signaling.^46^ Here, we examined whether the dysregulation of Ca^2+^ homeostasis was associated with SSd alterations in p75^NTR^ and TrkB signaling. Western blotting analysis showed that EGTA treatment significantly ameliorated the SSd‐induced disruption of m‐BDNF and pro‐BDNF protein expression in NPCs (Figure 6F), suggesting that [Ca^2+^]_i_ overload affected BDNF signaling. Furthermore, Figure 6G shows that EGTA treatment normalized the SSd‐induced reduction in p‐TrkB level, and Figure 6H shows that EGTA treatment also abolished the SSd‐induced activation of p75^NTR^ ICD but did not alter the FL of p75^NTR^. Taken together, these results indicated that the dysregulation of Ca^2+^ homeostasis was involved in SSd‐mediated p75^NTR^ and TrkB receptor signaling in NPCs.

### 3.7 SSd elevated Ca^2+^ concentration and disrupted neurotrophin receptor p75^NTR^/TrkB signaling in the hippocampus

**FIGURE 7:**
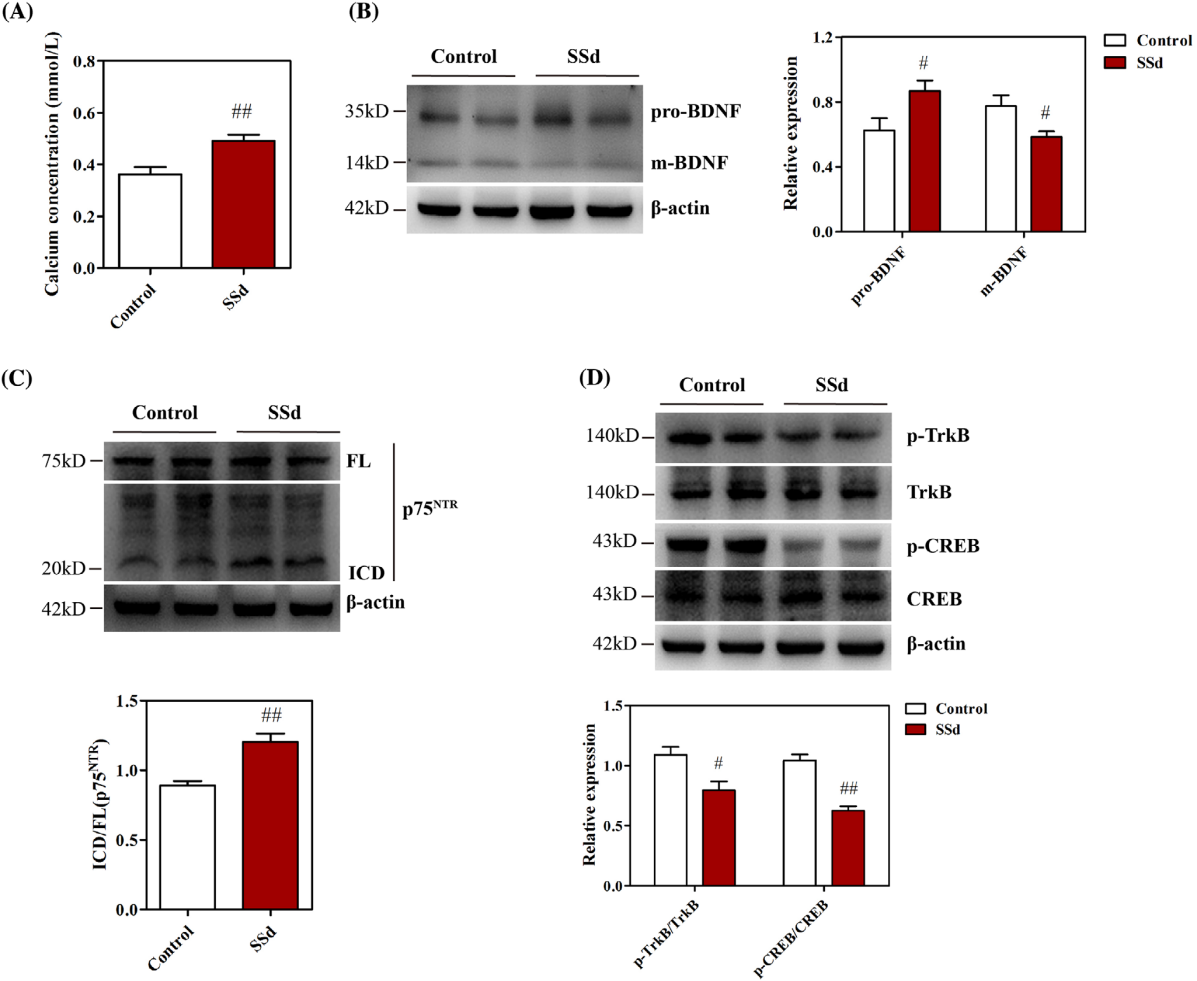
SSd elevated Ca^2+^ concentration and disrupted neurotrophin receptor p75^NTR^/TrkB signaling in the hippocampus. A, Effect of SSd on Ca^2+^ concentration in the hippocampus (n = 8). B, Western blot analysis for the effect of SSd on pro‐BDNF/m‐BDNF in the hippocampus and quantitative analysis of the levels of pro‐BDNF and m‐BDNF (n = 4). C, Western blot analysis of the effect of SSd on p75NTR activation in the hippocampus and quantitative analysis of the levels of p75^NTR^ ICD (n = 4). D, Western blot analysis for the effect of SSd on TrkB signaling in the hippocampus and quantitative analysis of the levels of p‐TrkB and p‐CREB (n = 4). Data are expressed as mean ± SEM. ^#^ *P *< .05 versus the control. ^##^ *P *< .01 versus the control

The hippocampal Ca^2+^ concentration was examined using a Ca^2+^ assay kit. As shown in Figure 7A, the Ca^2+^ concentration was significantly higher in SSd‐treated mice than in controls. Western blotting analysis showed that pro‐BDNF and m‐BDNF protein expressions were disrupted after SSd administration, resulting in the upregulation of pro‐BDNF and downregulation of m‐BDNF in the hippocampus of mice (Figure 7B). Treatment with SSd was also shown to increase the expression of p75^NTR^ ICD in mice (Figure 7C), suggesting that SSd induced activation of p75^NTR^ ICD in the hippocampus, consistent with the results of in vitro experiments. Moreover, as shown in Figure 7D, the administration of SSd inhibited TrkB signaling by suppressing the expression of p‐TrkB and p‐CREB in the hippocampus. These observations suggested that SSd elevated Ca^2+^ concentration and disrupted neurotrophin receptor p75^NTR^/TrkB signaling in the hippocampi of mice.

## 4 DISCUSSION

Evidence from clinical and preclinical studies has underlined the potential of cytostatic drugs to induce cognitive impairments.^47^ In the present study, we found that SSd treatment (16 mg/kg) caused impaired cognitive function and reduced adult hippocampal neurogenesis in mice. Our data were consistent with a previous report of a high incidence of cognitive dysfunction associated with impairment of hippocampal neurogenesis.^4^ Neurogenesis occurs in a series of tightly regulated steps, in which NPCs proliferate, survive, migrate, and differentiate into neurons or glia in the brain. In mouse primary hippocampal NPCs, we found that SSd suppressed cell proliferation and cell differentiation morphology of NPCs by induction of apoptosis, which decreased the survival of NPCs, suggesting that SSd exhibited neurotoxic effects both in mice and primary hippocampal NPCs. A previous study showed that SSd at a low dose of 1 mg/kg could ameliorate LPS‐induced neuroinflammation and attenuate neuronal apoptosis in mice,^48^ indicating that SSd may have a hormetic dose‐response and become toxic at increasing dose. Hormesis, which is defined as a biphasic response pattern characterized by a stimulatory effect at a low dose and inhibitory effect at a high dose, is an emerging area of pharmacological and toxicological research.^49^ Consistent with our observations, recent studies have shown that numerous plant‐derived agents exhibit hormesis and have demonstrated that a hormetic dose‐response is critical for neurological disorders, as well as for the accuracy of the therapeutic dose of pharmaceutical agents in human diseases.^50^, ^51^, ^52^, ^53^ These findings have implications with regard to the optimal dose range for future pharmacological investigations of SSd.

Neurotrophins and their receptors, Trk and p75^NTR^, play crucial roles in stem cell biology in the regulation of cell self‐renewal, survival, differentiation, and regeneration.^22^ As the co‐receptor of Trk, p75^NTR^ is attracting increasing attention as it exhibits the opposite function when stimulated, an interaction that is thought to preferentially lead to cell death by engaging with several downstream modulators.^13^, ^40^ The results of RNA‐Seq analysis in the present study indicated that NRAGE, NRIF, and NADE, well‐identified factors that promote p75^NTR^‐dependent apoptosis signaling, were enriched in SSd‐treated mice, which prompted us to evaluate the apoptosis of NPCs and explore p75^NTR^ signaling. Further experiments suggested that SSd led to apoptosis in the hippocampus and primary NPCs through JNK induction and caspase activation, as well as activated p75^NTR^ ICD. In parallel with our findings, there is accumulating evidence that p75^NTR^ induces apoptosis in sensory neurons,^54^ sympathetic neurons,^55^ Schwan cells,^56^ and hippocampal neurons.^57^ In vivo studies showed a marked increase in neuronal apoptosis in mice overexpressing p75^NTR^ ICD, and this was reversed in the retina in mice lacking the gene encoding p75^NTR^.^58^, ^59^ However, another study showed that mice with knockout of p75^NTR ExIII^, lacking the FL receptor p75^NTR^, exhibited decreased numbers of neuroblasts and newborn neurons in the DG, as well as increased cell death.^60^ These contradictory results may be explained by the levels of the different domains of p75^NTR^. It has been suggested that the receptor will show pro‐survival functional signaling when p75^NTR^ ICD lacks catalytic activity, while the release of p75^NTR^ ICD by extracellular cleavage could result in neuronal cell death,^61^ indicating that p75^NTR^ ICD rather than the FL receptor may be closely involved in downstream signaling leading to apoptosis or survival. This was further supported by a report that p75^NTR ExIV−/−^ mice expressed the p75^NTR^ ICD, overexpression of which causes apoptosis cascades.^37^ Consistent with the findings, our results showed that SSd upregulated the level of p75^NTR^ ICD without changing p75^NTR^ FL. Moreover, shRNA targeting p75^NTR^ ICD effectively inhibited the activation of p75^NTR^ ICD under conditions of SSd treatment and ameliorated the SSd‐induced increase in activity of the NRAGE/NRIF/NADE/JNK cell apoptotic pathway, followed by attenuated survival of NPCs, which subsequently ameliorated SSd‐induced inhibition of NPC proliferation and differentiation. These observations indicated that SSd‐induced p75^NTR^ apoptotic signaling contributed to the neurotoxic effect on NPCs.

It is well known that p75^NTR^ is activated by neurotrophins and is more effectively activated by pro‐neurotrophin. There have been many studies of the involvement of neurotrophins in adult neurogenesis, in particular, BDNF is one such factor that has been demonstrated to be important in hippocampal neurogenesis. Previous research has demonstrated that pro‐BDNF could cause neuronal apoptosis by inducing p75^NTR^ pathogenicity and a decrease of trophic effects in patients with Alzheimer's disease,^13^ suggesting that pro‐BDNF neurotoxic signaling is associated with stimulation of p75^NTR^ ICD. This was supported by the results of the present study indicating that SSd upregulated the levels of pro‐BDNF, suggesting that SSd‐activated p75^NTR^ ICD may be related to pro‐BDNF signaling. Moreover, mice with BDNF depletion were reported to show markedly decreased survival of adult‐born granule cells.^62^ Adult neurogenesis was inhibited by BDNF knockdown in the DG but increased by the injection of exogenous BDNF in rodents.^7^, ^8^ Meanwhile, BDNF was shown to be beneficial for the survival of hippocampal NPCs depending on TrkB signaling.^9^ Accordingly, BDNF appears to be essential for neurogenesis by binding to TrkB to trigger neurotrophic signaling. Consistent with these observations, our results showed that SSd downregulated the levels of m‐BDNF and TrkB/CREB signaling both in mice and in primary NPCs, the effects of which could be attenuated by the addition of recombinant BDNF, suggesting that a decrease in trophic effects is involved in SSd‐induced damage to hippocampal NPCs. Neurotrophins other than BDNF, such as NGF, have also been shown to be associated with neurogenesis. There is evidence that NGF exhibits a synergistic effect on the proliferation and survival of NPCs, which contributes to improved neurological function.^63^ Nevertheless, we found that NGF signaling was not significantly changed in the hippocampus of SSd‐treated mice (not shown), indicating that the SSd administration did not affect NGF signaling.

As a potent Ca^2+^ ionophore, SSd has been shown to trigger elevation of [Ca^2+^]_i_ via a nonselective mechanism.^24^ The cellular Ca^2+^ overload, or intracellular Ca^2+^ dysregulation, can be caused by both pathological processes and toxic agents, and hence induces cytotoxicity resulting in apoptotic cell death. Of particular interest is that the intracellular Ca^2+^ concentration subserves complex signaling roles in the brain, which are involved in neural plasticity underlying learning and memory.^64^ Our results showed that SSd increased the Ca^2+^ levels in the hippocampus and induced [Ca^2+^]_i_ overload in NPCs, which could be ameliorated by the extracellular Ca^2+^ chelator EGTA rather than intracellular Ca^2+^ chelator BATPA/AM. Moreover, EGTA also attenuated the viability of NPCs exposed to SSd, suggesting that SSd induced cytotoxicity in NPCs through a Ca^2+^‐mediated mechanism. This nonselective ionophore activity of SSd may account for its cytotoxicity to both cancer cells and primary cells.^24^, ^65^ Ca^2+^ homeostasis plays an essential role in modulating BDNF signaling through protein kinase cascades that lead to the activation of several transcription factors.^46^, ^66^ Therefore, we investigated whether Ca^2+^ signaling was involved in SSd‐disordered signaling in NPCs, and the results showed that restoring the Ca^2+^ homeostasis by EGTA could mediate the BDNF/TrkB and pro‐BDNF/p75^NTR^ signals and attenuate SSd‐induced damage in NPCs. The above results indicate that cytoplasmic Ca^2+^ signaling is related to the neurotoxic effects of SSd on NPCs and neurotrophic receptor signals. As SSd may show a hormetic dose‐response, this raises questions about the molecular biological mechanism, specifically, whether the effects observed at a low dose will be affected at a high dose, showing a corresponding toxic reaction. There is accumulating evidence that SSd can alleviate oxidative stress and have protective effects in some models both in animals and in cells, while a relatively high dose of SSd was also suggested to induce oxidative stress leading to pathological injury in mice,^25^ suggesting that redox status may be involved in the mechanism of hormesis of SSd. Redox homeostasis has attracted increasing attention for its crucial role in neurodegenerative disorders,^67^, ^68^ and neuroscience studies have reported that increased cytoplasmic Ca^2+^ is associated with excessive levels of reactive oxygen species,^64^ the derived advanced glycation end products of which were demonstrated to block the process of transformation from pro‐BDNF to m‐BDNF in the hippocampus,^13^ Therefore, further investigations are required to examine whether oxidative stress is involved in SSd‐induced dysfunction of neurotrophic receptor signaling.

Our findings have strong translational implications for the optimal dose range of SSd in further pharmacological research. However, this study had some limitations. Compared with the definite adult hippocampal neurogenesis that has been confirmed in rodents and other mammals, human adult hippocampal neurogenesis remains controversial. Several early studies demonstrated that adult neurogenesis occurred in the human DG.^69^, ^70^ However, one study reported that hippocampal neurogenesis, although present in children, was not persistent or was extremely rare in adults.^3^ Interestingly, a recent study showed that hippocampal neurogenesis persisted in neurologically healthy people up to the ninth decade of life and dropped sharply in patients with cognitive impairment.^4^ Differences in the methodologies used to detect neural markers with immunohistochemical techniques may be largely responsible for the controversy and raise questions and challenges about how to study adult neurogenesis in the human brain. Some groups have pointed out that dealing with human samples has unique problems, and the results of neurogenesis in animals cannot be simply translated to humans due to phylogenetic differences.^71^, ^72^ Therefore, more complete analysis and standardized methodologies are required to investigate neurogenesis under both physiological and pathological conditions, such as single‐cell RNA‐Seq or other methods of following the process of adult neurogenesis in living tissue.

**FIGURE 8:**
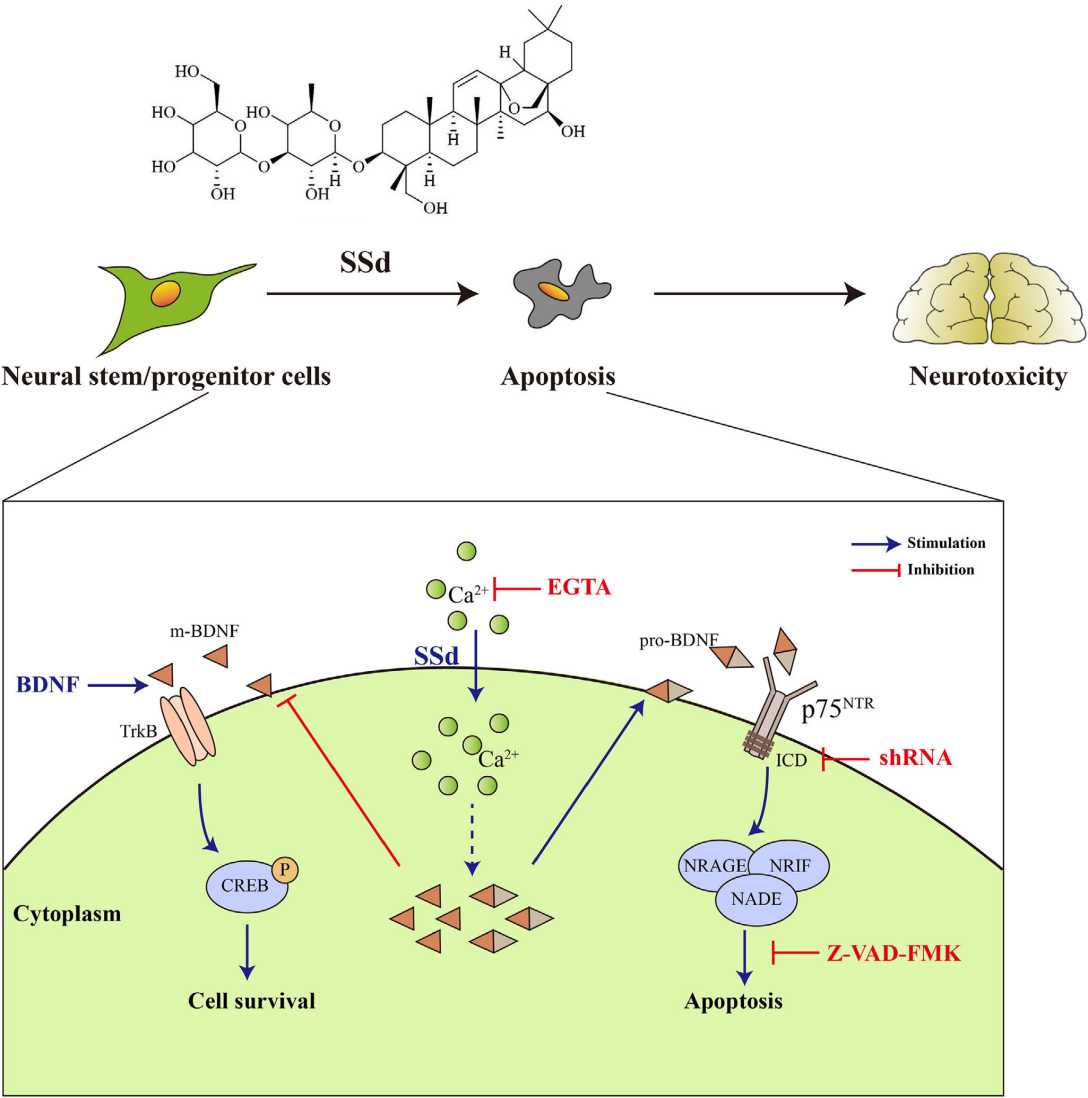
The schematic presentation indicates the suggested mechanisms by which SSd induces cognitive dysfunction through inhibiting the survival of hippocampal NPCs. This neural damage is through the activation of p75^NTR^ cell death signaling and inhibition of TrkB signaling, engaged by disordered BDNF pathway dependent on the dysregulation of cellular Ca^2+^ homeostasis

In summary, the results of the present study indicated that SSd contributes to cognitive dysfunction by impairing hippocampal neurogenesis via inhibition of the survival of NPCs in mice. A comprehensive mechanism for SSd‐induced neurotoxicity is shown in Figure 8. These results present a neurological basis for SSd‐induced neurotoxicity, which contributes to a better understanding of the neurotoxic effects of cytostatic drugs or Ca^2+^ ionophores and has implications for the further pharmacological investigation of SSd.

## CONFLICT OF INTEREST

The authors declare that there is no conflict of interest that could be perceived as prejudicing the impartiality of the research reported.

## AUTHOR CONTRIBUTIONS

Tingting Qin, Ziqiao Yuan, Zhanqiang Ma, and Shiping Ma participated in the research design. Tingting Qin, Ziqiao Yuan, Jiayu Yu, and Xinxin Fu carried out the experiments. Tingting Qin, Ziqiao Yuan, Xueyang Deng, and Qiang Fu analyzed the data. Tingting Qin and Ziqiao Yuan wrote the manuscript. Zhanqiang Ma and Shiping Ma assisted in writing. All authors read and approved the final manuscript.

## ETHICAL APPROVAL

Animal care was carried out following the provision and general recommendation of the Chinese Experimental Animals Administration Legislation. The procedure was approved by the Ethics Committee of China Pharmaceutical University and the Laboratory Animal Management Committee of Jiangsu Province. This manuscript does not contain patient data or clinical studies.

**Abbreviations**
[Ca^2+^]_i_: cellular concentration of Ca^2+^
BDNF: brain‐derived neurotrophic factor
BrdU: bromodeoxyuridine
Ca^2+^: calcium
DCX: Doublecortin
DG: dentate gyrus
EdU: 5‐ethynyl‐2′‐deoxyuridine
FBS: fetal bovine serum
FI: fluorescence intensity.
GSEA: gene set enrichment analysis
ICD: intracellular domain
JNK: c‐Jun N‐terminal kinase
MOI: multiplicity of infection
NPCs: neural stem/progenitor cells
P75^NTR^: p75 neurotrophin receptor
PBST: Triton X‐100 in PBS
PFA: paraformaldehyde
PLL: poly‐L‐lysine
RGLs: radial glia‐like cells
SSd: saikosaponin‐d
TrkB: tyrosine kinase receptor B

## Supporting information

SUPPORTING INFORMATION

## Data Availability

The data used to support the findings of this study are available from the corresponding author upon request.
