# Supplementary material for: Saikosaponin‐d impedes hippocampal neurogenesis and causes cognitive deficits by inhibiting the survival of neural stem/progenitor cells via neurotrophin receptor signaling in mice
Source: Clin Transl Med. 2020 Dec 21;10(8):e243. doi: 10.1002/ctm2.243 (PMC7752162; doi:10.1002/ctm2.243)
Supplement: Supplementary file 1 — SUPPORTING INFORMATION [file CTM2-10-e243-s001.docx]

**
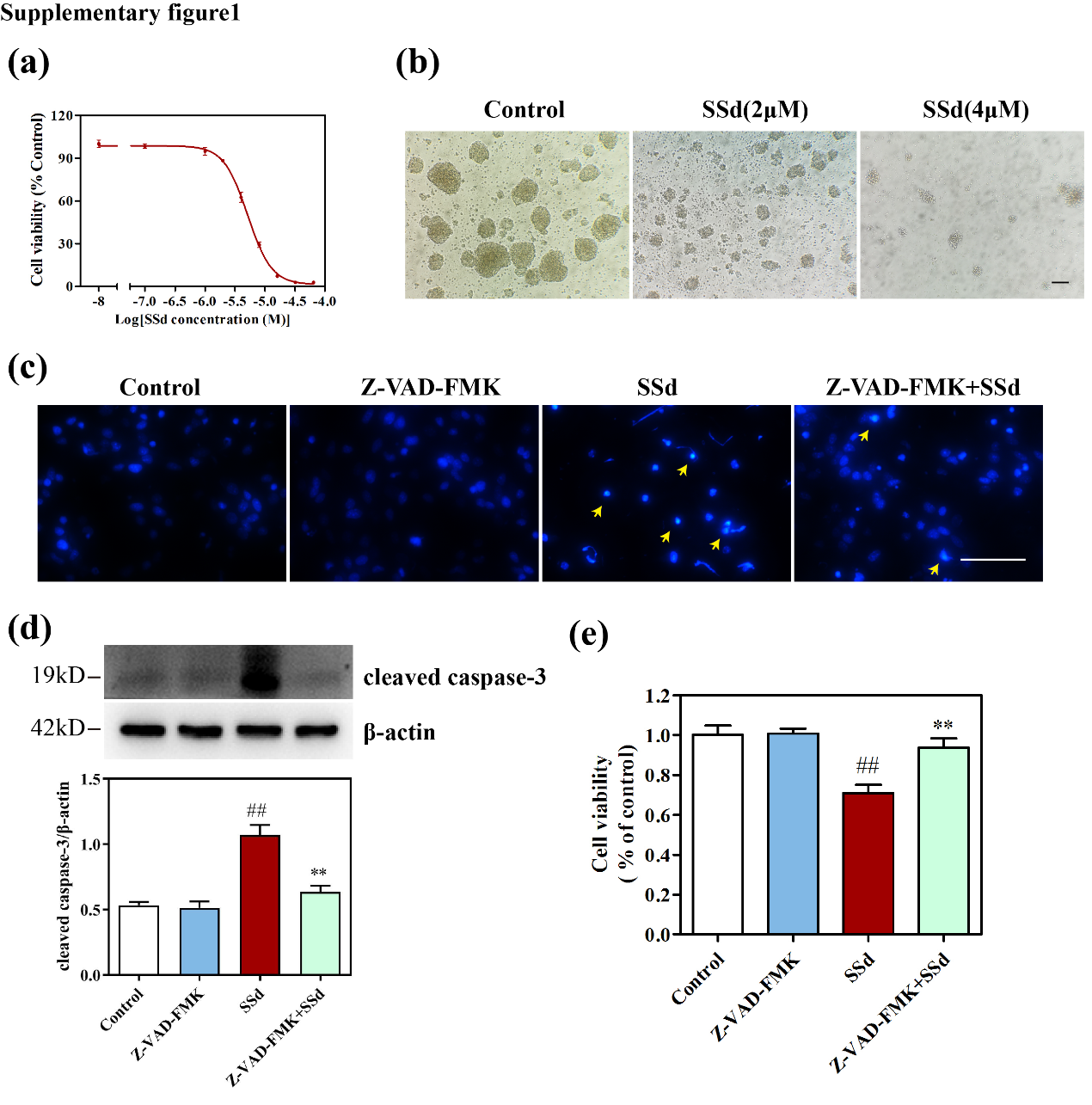
**

**Figure S1 SSd reduced the survival of NPCs through the apoptotic pathway.** (a) Effect of SSd on cell viability. The NPCs were treated with different concentrations of SSd for 24 hours, and the cell viability was detected by MTT assay. (b) Effect of SSd on neurospheres growth. The neurospheres were seeded in the 6-well plate and treated with SSd for 24 hours. Images were obtained from the microscope. Scale bar 100μm. (c) Effect Z-VAD-FMK on SSd-induced apoptosis in NPCs. The NPCs were treated with Z-VAD-FMK in the absence or presence of SSd for 24 hours and cell apoptosis was detected using Hoechst 33342 staining. Scale bar 25μm. (d) Western blot analysis of the effect of Z-VAD-FMK on SSd-induced levels of cleaved caspase-3 and quantitative analysis of the levels of cleaved caspase-3. (e) Effect of Z-VAD-FMK on SSd-inhibited cell viability. The NPCs were treated with Z-VAD-FMK in the absence or presence of SSd for 24 hours, and the cell viability was measured by MTT assay. Data were expressed as mean ± SEM. The results are representative of 3 independent experiments. ^##^*p* < 0.01 *vs*. the control; ^**^*p* < 0.01 *vs.* SSd.
